# Crystal Structures and Microwave Dielectric Properties of Novel MgCu_2_Nb_2_O_8_ Ceramics Prepared by Two-Step Sintering Technique

**DOI:** 10.3390/ma15228053

**Published:** 2022-11-15

**Authors:** Sen Peng, Chen Li, Chao Tang, Sheng Liu, Shengxiang Huang, Leilei Qiu, Lianwen Deng

**Affiliations:** 1 School of Physics and Electronics, Central South University, Changsha 410083, China; 2 Provincial Key Laboratory of Informational Service for Rural Area of Southwestern Hunan, Shaoyang University, Shaoyang 422000, China; 3 College of Mechanical Engineering, Hunan Institute of Engineering, Xiangtan 411104, China

**Keywords:** MgCu_2_Nb_2_O_8_ ceramics, crystal structure, annealing, microwave dielectric property

## Abstract

In this work, novel MgCu_2_Nb_2_O_8_ (MCN) ceramics were synthesized by the two-step sintering (TSS) technique, and the phase composition, crystal structures, and microwave dielectric properties were comprehensively studied. X-ray diffraction (XRD) and Raman analysis demonstrated that MCN ceramics are multi-phase ceramics consisting of MgNb_2_O_6_ and CuO phases. X-ray photoelectron spectroscopy (XPS) was utilized to investigate the chemical composition and element valence of MgCu_2_Nb_2_O_8_ ceramics. Scanning electron microscopy (SEM) analysis demonstrated dense microstructures in the MCN ceramics prepared at a sintering temperature of 925 °C. The microwave dielectric properties were largely affected by the lattice vibrational modes and densification level of the ceramics. The outstanding microwave dielectric properties of ε_r_ = 17.15, *Q* × *f* = 34.355 GHz, and τ*_f_* = −22.5 ppm/°C were obtained for the MCN ceramics sintered at 925 °C, which are results that hold promise for low temperature co-fired ceramic (LTCC) applications.

## 1. Introduction

Microwave dielectric ceramics (MWDC) are usually used as dielectric materials in the microwave frequency band (including UHF and SHF frequency band, 300 MHz~300 GHz), and they can be used to perform a variety of functions [1,2]. They are new functional ceramic materials developed in the last 20 years and are also the critical material for the production of microwave resonators and dielectric filters [3,4,5]. Microwave dielectric ceramics have attracted great attention because of their exceptional features, such as their high-quality, low microwave loss, and stable temperature performance [6,7]. Based on the original microwave ferrite, many researchers have greatly improved the formula and manufacturing process of microwave dielectric ceramics [8]. Microwave dielectric ceramics are suitable for manufacturing modern products, including navigation, communication, radar, household satellite TV receivers, and mobile phones. In pursuit of microwave circuits with high integration and reliability, they can also be used to make oscillators, filters, and frequency discriminators [9,10]. Along with the rapid development of microwave technology, much attention has been paid to studying microwave dielectric ceramics, which hold much potential for the future of microwave devices.

Along with the fast-growing development of fifth-generation mobile communication (5G), microwave ceramic filters have come to play a major role in the 5G base station filter due to their superior dielectric properties, which in turn has resulted in higher requirements for microwave dielectric ceramics [11,12]. Niobate ceramics such as RNb_2_O_6_ (R = Ca, Co, Cu, et al.) [13], R_3_Nb_2_O_8_ [14], RMNb_2_O_8_ (M = Zr, Ti) [2], ATiNbO_6_ (A = rare earth) [15], and ANbO_4_ [16], have long been widely and deeply studied because of their excellent microwave properties. Furthermore, the novel copper–niobate ACu_2_Nb_2_O_8_ (A = Ni, Ca, Mg, Co, or Zn) ceramics have attracted great attention because of their optimal dielectric properties and low sintering temperature (S_T_) for LTCC applications [17]. Yang [18] reported CaCu_2_Nb_2_O_8_ ceramics that contained several phases and that showed outstanding microwave dielectric properties of ε_r_ = 15.7, *Q* × *f* = 28.700 GHz and τ*_f_* = −38.4 ppm/°C, which are promising results for LTCC applications. MgCu_2_Nb_2_O_8_ ceramics are less-studied, especially for their single-phase or multi-phase structure. We proposed the two-step sintering technique to prepare MgCu_2_Nb_2_O_8_ microwave ceramics with optimal dielectric properties and investigated their phase composition, microstructure, and microwave dielectric characteristics. In this paper, the effects of synthesis conditions on phase composition, microstructure, crystal structure and microwave dielectric properties of MCN ceramics were carefully analyzed, and our results provide a valuable reference for MCN ceramics researchers.

## 2. Experimental

MgCu_2_Nb_2_O_8_ ceramics were synthesized by the solid-phase reaction process with the use of high-purity oxides of MgO (98%), CuO (99%), and Nb_2_O_5_ (99.9%) produced by Shanghai Aladdin Biochemical Technology Co., Ltd. (Aladdin, Shanghai, China). First, the raw materials were mixed and ball-milled with deionized water and zirconia balls for 24 h. Then, the mixtures were calcined at 750 °C for 5 h and re-milled for 24 h. After this, the calcined powders were mixed with 6 wt% polyvinyl alcohol as a binder and pressed into cylinders of 15 mm × 7 mm. Finally, the pressed samples were sintered using the two-step sintering technique. The samples were initially sintered at temperatures between 875 °C and 1000 °C for 10 h to obtain an intermediate density, and then they were annealed at 800 °C for 4 h to obtain high density.

The phase composition was checked by X-ray diffraction (Aolong, AL-2700B, Dandong, China) with copper Kα radiation. The microstructure was analyzed via scanning electron microscope (Hitachi, S4800, Tokyo, Japan). The crystal structure of the MgCu_2_Nb_2_O_8_ ceramics was verified through Raman spectroscopy (Renishaw, London, UK, 532 nm). The element valence state in the MgCu_2_Nb_2_O_8_ ceramics was studied via X-ray photoelectron spectroscopy (VG Scientific, ESCALAB 250, Waltham, MA, USA). The apparent density of the ceramic system was acquired using the Archimedes method. The porosity and average grain size (AG) were estimated using ImageJ software (ImageJ.JS). A network analyzer (Agilent, N5230A, Santa Clara, CA, USA) was performed on the MCN ceramics to attain microwave dielectric properties over a temperature range of 25–85 °C [19].

## 3. Results and Discussion

**Figure 1:**
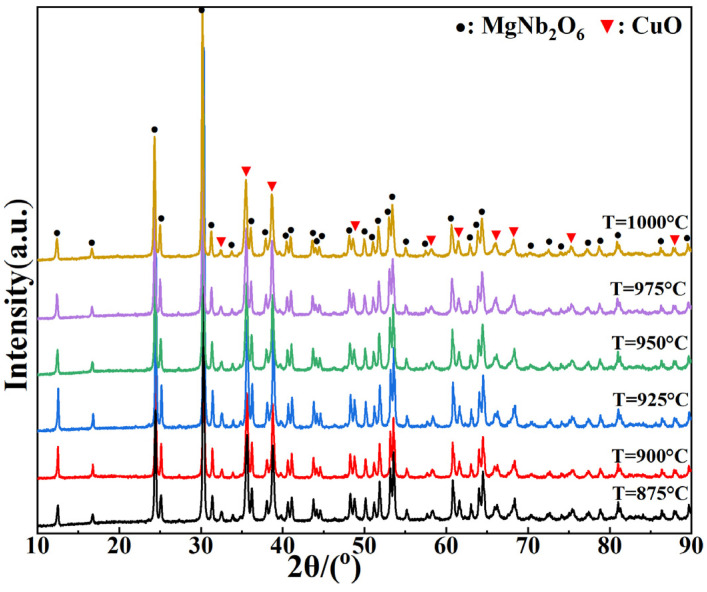
XRD patterns of MgCu_2_Nb_2_O_8_ ceramics sintered at different temperatures.

The XRD patterns of MgCu_2_Nb_2_O_8_ ceramics sintered at 875–1000 °C are shown in Figure 1. The MgNb_2_O_6_ phase (JCPDS #88–0708) was observed from the XRD analysis, and its structure was identified as the orthorhombic columbite-type structure in space group Pbcn [20]. Meanwhile, the diffraction peaks of CuO (JCPDS #48–1548) were also detected and identified as the monoclinic structure with space group C2/c [21]. Careful examination of the XRD patterns indicated that no other phases existed except these two phases. Therefore, it could be inferred that the MgCu_2_Nb_2_O_8_ ceramics are multi-phase ceramics consisting of MgNb_2_O_6_ and CuO phases.

**Figure 2:**
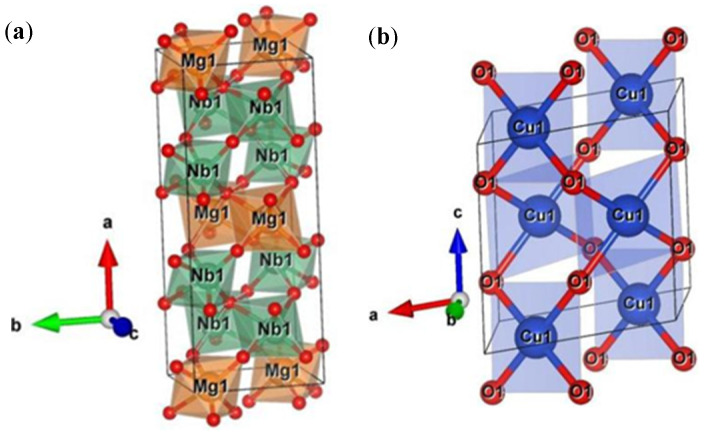
The crystal structures of (**a**) orthorhombic columbite MgNb_2_O_6_ and (**b**) monoclinic CuO.

**Figure 3:**
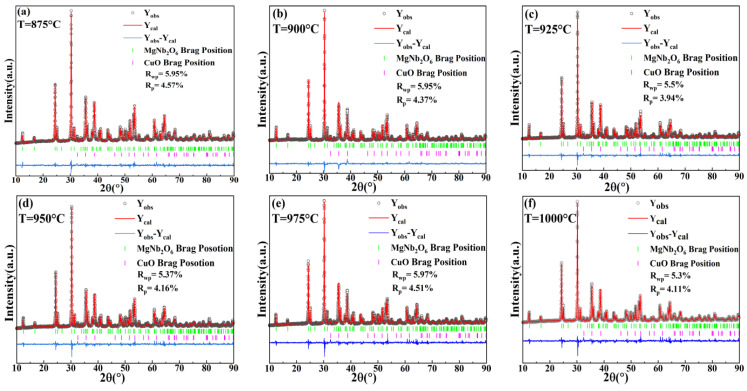
Rietveld refinement patterns of MgCu_2_Nb_2_O_8_ ceramics sintered at several temperatures from 875 °C to 1000 °C.

The Rietveld refinement was performed to further explore the structural characteristics of MgCu_2_Nb_2_O_8_ ceramics, and the refined cell parameters of MgCu_2_Nb_2_O_8_ ceramics sintered at the temperatures from 875 °C to 1000 °C were obtained by the GSAS software. The orthorhombic columbite MgNb_2_O_6_ and monoclinic CuO were introduced as individual phases in the refinement model, and their specific crystal structures are exhibited in Figure 2. Figure 3 displays the refinement patterns of MgCu_2_Nb_2_O_8_ ceramics sintered at different temperatures in the range from 875 °C to 1000 °C. The refinement XRD patterns are in good agreement with the measured results, demonstrating that MgCu_2_Nb_2_O_8_ ceramics are the multi-phase ceramics composed of MgNb_2_O_6_ and CuO phases. The crystallographic parameters and reliability factors of MgCu_2_Nb_2_O_8_ ceramics, including profile factors (R_p_), weighted profile factors (R_wp_), and goodness of fit values (*χ*^2^), are listed in Table 1. The results showed that all R_p_ and R_wp_ values obtained using GSAS software are below 10% and R_p_ is less than R_wp_, suggesting that the refinement data obtained by GSAS software are reliable.

**Figure 4:**
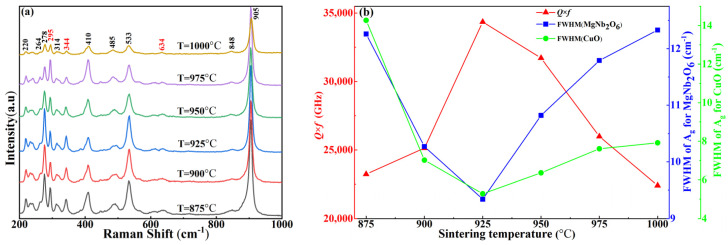
(**a**) The Raman spectra of MgCu_2_Nb_2_O_8_ ceramics; (**b**) The correlations between *Q* × *f* values and FWHM values.

Raman phonon modes can be used not only to analyze the crystal structure, but also to obtain the phase composition of the ceramic system. The Raman spectra of MgCu_2_Nb_2_O_8_ ceramics sintered at different temperatures are shown in Figure 4a. Twelve Raman modes, located at 220, 264, 278, 295, 314, 344, 410, 485, 533, 634, 848 and 905 cm^−1^, were detected. As for the MgNb_2_O_6_ phase reported by Wu [20], the weak peak at 220 cm^−1^ is attributed to the O-Nb-O bending mode. The bands at 250–400 cm^−1^ are identified as the twisting vibration of octahedron. The modes located at 410, 485, 533, 848 and 905 cm^−1^ are assigned as the stretching vibration of Nb-O bonds. For CuO, three Raman active optical phonons (Ag +2Bg) could be observed clearly from the Raman spectra. The peaks of 295, 344 and 634 cm^−1^ are correspondent to the Ag, Bg and Bg modes, respectively, and it is consistent with the previous results [21]. Thus, Raman analysis also proves that MgCu_2_Nb_2_O_8_ ceramics are composed of MgNb_2_O_6_ and CuO. In addition, Raman phonon modes are sensitive to not only the crystal structures but also to dielectric properties of the ceramics. As presented in Figure 4b for the A_g_(MgNb_2_O_6_ or CuO) modes, the full width at half-maximum (FWHM) displays an opposite changing trend as that in *Q* × *f* value. As reported by Liu [22], FWHM value is closely related to the damping coefficient, which had a great influence on the dielectric losses. Generally, a weaker FWHM value usually corresponded to a higher *Q* × *f* value. Notably, the sample sintered at 925 °C possessed the weakest FWHM and highest *Q* × *f* value, which was completely consistent with the results of ref. [22].

**Figure 5:**
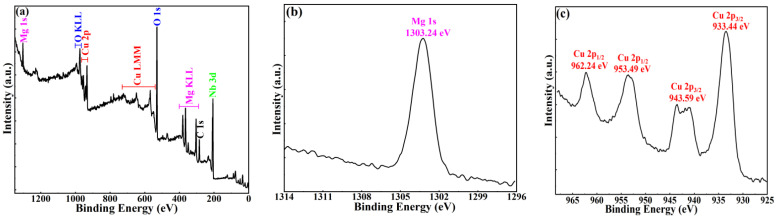

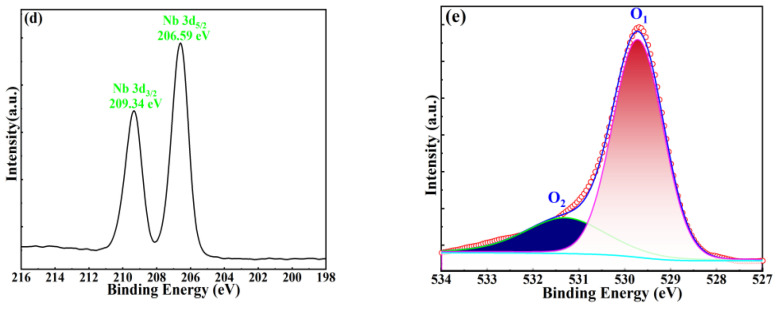
XPS spectra of MgCu_2_Nb_2_O_8_ ceramics sintered at 925 °C: (**a**) the survey spectrum, (**b**) Mg 1s, (**c**) Cu 2p, (**d**) Nb 3d, and (**e**) O 1s.

The chemical composition and element valence of MgCu_2_Nb_2_O_8_ ceramics were studied by XPS analysis, and the resulted spectra are given in Figure 5. The survey XPS spectrum, as shown in Figure 5a, displays the presence of Mg 1s, Cu 2p, Nb 3d, O 1s, O KLL, Cu LMM, Mg KLL and C 1s, and it verifies the chemical purity of the MCN ceramics. The XPS data obtained from the sample were all corrected for charging effects with reference to the C1s peak fixed at 284.8 eV. Figure 5b shows the narrow scan XPS spectrum of Mg 1s for MCN ceramics. The peak of Mg 1s was at 1303.24 eV, corresponding to the characteristic spectrum of Mg with a valence of +2 [23]. The Cu 2p spectrum, as shown in Figure 5c, presented spin-orbit components of Cu 2p_1/2_ and Cu 2p_3/2_ at 962.24, 953.49, 943.59 and 933.44 eV, which were assigned to Cu^2+^ [24]. The narrow scan XPS spectrum of Nb 3d for MCN ceramics is exhibited in Figure 5d, consisting of spin-orbit doublet peaks Nb 3d_3/2_ and Nb 3d_5/2_ at 209.34 and 206.59 eV (Δ = 2.75 eV), respectively, which is in good agreement with the characteristic spectra of Nb^5+^ in XPS [25,26]. The peak of O1s (O_1_) for MCN ceramics shown in Figure 5e was at 529.69 eV, which was indexed to O^2−^ [24,27]. In addition, it could be found that, besides the main peak, a shoulder (O_2_) at 531–533 eV is evident in the O 1s core level and this component can be attributed to the hydrocarbonates formation at the sample surface [26]. The above analysis proved that the chemical states of Cu, Mg, Nb and O were +2, +2, +5 and −2, respectively.

**Figure 6:**
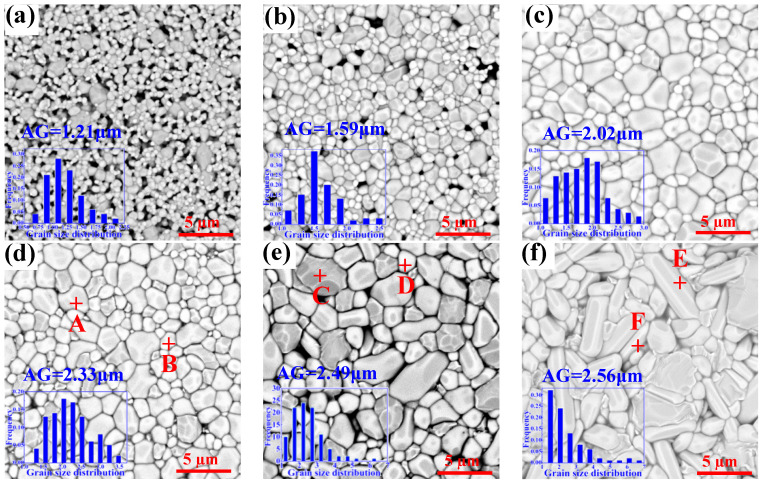
SEM images and grain size distribution (inset figure) of MgCu_2_Nb_2_O_8_ ceramics sintered at (**a**) S_T_ *=* 875 °C, (**b**) S_T_ *=* 900 °C, (**c**) S_T_ *=* 925 °C, (**d**) S_T_ *=* 950 °C, (**e**) S_T_ *=* 975 °C, and (**f**) S_T_ *=* 1000 °C.

Figure 6 is the SEM images recorded for the MgCu_2_Nb_2_O_8_ ceramics sintered at different temperatures in the range from 875 °C to 1000 °C. As observed in Figure 6a, the sample possessed a porous microstructure, and the average grain size is about 1.2 μm, indicating low density and insufficient sintering. It could be seen From Figure 6b, the grain growth is obvious, and the average grain size reached 1.59 µm. While, there were also many small grains in dense contact and the pores were eliminated gradually as compared to Figure 6a. As S_T_ increased gradually to 925 °C, the samples presented compact microstructures with clear grain boundaries. In addition, the grain growth was further enhanced and some abnormal grains were obvious, meanwhile, the average grain size was increased to 2.02 µm. In general, migration and diffusion of ions were beneficial to the grain growth at the proper sintering temperature. It is proved that the densification process of ceramic system by traditional solid-state ceramic route is largely determined by the migration and diffusion of ions, and proper sintering temperature could assist these processes [28]. Thus, the grain growth was enhanced gradually as S_T_ increased. With further increasing S_T_, the abnormal grains in the samples become more than before, and it yields strong negative effects on the densification.

In order to further determine compositions of the abnormal grains, EDS was developed to explore the large grains (spots A, C and E) and small grains (spots B, D and F), as shown in Table 2. Notably, spots A, C and E in the large grains displayed Mg:Nb:O elemental molar ratios of 11.81:22.38:65.81, 11.93:22.66:65.41 and 12.06:23.71:64.23 respectively, which are close to that in MgNb_2_O_6_. And the elemental molar ratios of Cu:O in the small grains (spots B, D and F) were 52.12:47.88, 50.87:49.13 and 51.59:48.41 respectively, similar to that in CuO. Combining with the XRD patterns shown in Figure 1, it turned out that the large grains are MgNb_2_O_6_ and the small grains are CuO.

The relations between the apparent density, relative density and porosity of the MCN ceramics sintered at the temperatures from 875 °C to 1000 °C can be observed in Table 3, which indicates that the apparent density and relative density first increase and then decrease with an increase of sintering temperature, while the porosity shows an opposite varying trend as compared to that of apparent density. Moreover, the sample prepared at the sintering temperature of 925 °C presented the largest apparent density of 5.519 g/cm^3^, corresponding to the smallest value of 1.13% for porosity. With the increase of S_T_, the apparent density firstly increased to the peak value of 5.519 g/cm^3^ at S_T_ = 925 °C, which was primarily accused to the improvement of densification caused by the appropriate sintering temperature, as shown in Figure 6c. However, when S_T_ > 925 °C, with the further increase of S_T_, the apparent density showed a downward trend and accompanied by the increase of porosity. Thus, combining with Figure 6d–f, this phenomenon was due to the loose microstructures caused by the formation of abnormal grain. In general, porosity greatly affected the apparent density of MCN ceramics, which might be attributed to the dense and uniform microstructure [29].

**Figure 7:**
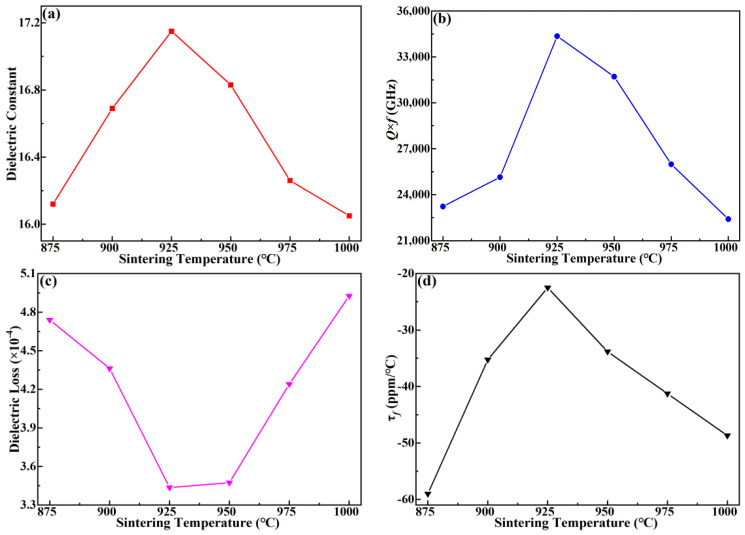
Microwave dielectric properties of MgCu_2_Nb_2_O_8_ ceramics sintered at different temperatures: (**a**) ε_r_, (**b**) *Q* × *f*, (**c**) τ*_f_*, and (**d**) tanδ.

Figure 7 presents the dielectric constant (ε_r_), quality factor (*Q* × *f*), temperature coefficient of resonant frequency (τ*_f_*), and dielectric loss (tanδ) of the MgCu_2_Nb_2_O_8_ ceramics sintered at the temperatures in the range from 875 °C to 1000 °C. Careful examination of the curves reveals that ε_r_ displays a homologous varying trend as that of apparent density shown in Table 3. Generally speaking, the ε_r_ is mainly dependent on the phase composition and densification of the samples [22]. In this study, ε_r_ starts to increase initially and reaches the peak value of 17.15 at 925 °C, which is attributed to the enhancement of densification caused by the appropriate sintering temperature. To an extent, the densification of sintered ceramics has a dominant role to increase the dielectric constant, which might be ascribed to the dense and uniform microstructure at high densities. At S_T_ > 925 °C, the dielectric constant starts to decline, and it is due to the decrease of densification caused by the expansion in abnormal grains. Combining the results shown in Figure 6 and Figure 7a, the expansion in abnormal grains should be responsible for the decline in dielectric constant.

Figure 7b,c display the correlations between *Q* × *f* value and dielectric loss (tanδ) in the sintered MgCu_2_Nb_2_O_8_ ceramics. Analysis on the *Q* × *f* and tanδ indicated that the *Q* × *f* value shows an opposite varying trend as that of tanδ. As for *Q* × *f* value, it first increase to the peak value of 34.355 GHz at S_T_ = 925 °C, and then declines with the increase of S_T_. As is well known, the *Q* × *f* value is influenced by a lot of factors, such as dielectric loss, densification, crystal defects, second phases and the average grain size [28,30]. Moreover, dielectric loss is not only related to intrinsic loss because of the lattice vibration mode, but extrinsic loss, including cation ordering degree, grain size, second phases and defects [31]. When 875 °C ≤ S_T_ ≤ 925 °C, the *Q* × *f* value presents an upward trend. For one thing, the increase is derived from the decreased intrinsic loss owing to the deteriorated FWHM value, as shown in Figure 4b. For another, the increased densification caused by the proper sintering temperature is beneficial to the enhancement of *Q* × *f* value. It was clear that the *Q* × *f* value increases from 23.230 GHz to 34.355 GHz when the microstructures get improved, and dense microstructure generally corresponds to higher *Q* × *f* value [32]. While the decline in *Q* × *f* value was attributed to the increased intrinsic loss and poor densification due to the abnormal grain grown. As depicted in Figure 7c, the dielectric loss declined firstly, and acquired the minimum value of 3.436 × 10^−4^ at S_T_ = 925 °C, then started to increase with increasing S_T_, showing an opposite varying trend as that of *Q* × *f* value. Combining with Figure 6 and Figure 7, it could be seen that the *Q* × *f* value was primarily determined by the dielectric loss and compactness of sintered ceramics [33,34]. Careful examination of the Figure 7 presented that proper sintering temperature led to the elevation of *Q* × *f* values from 23.230 GHz to 34.355 GHz as well as the decrease of dielectric loss from 4.740 × 10^−4^ to 3.436 × 10^−4^. Therefore, the increased densification had a dominant role to improve *Q* × *f* value and reduce dielectric loss. Furthermore, the two-step sintering technique was conducive to reducing the internal stress during the sintering process, and thus improving the *Q* × *f* value [35]. Moreover, the two-step sintering technology could enable microwave dielectric ceramics to be densified at a temperature lower than the normal sintering temperature, and obtained excellent dielectric properties [11]. Therefore, due to the advantages of two-stage sintering technology, we improved the sintering scheme properly in this work to attenuate the internal stress under the condition of obtaining high-density ceramic samples. In fact, in this work, the two-step sintering technique has greatly improved the microwave dielectric properties of the samples. As presented in Figure 7b, the *Q* × *f* value of the sintered ceramics was significantly improved, reaching the maximum value of 34.355 GHz.

The τ*_f_* values of MgCu_2_Nb_2_O_8_ ceramics sintered at the temperature range from 875 °C to 1000 °C are ascribed in Figure 7d. It is deserved to note that the τ*_f_* value initially increases to −22.5 ppm/℃ at S_T_ = 925 °C, and then declines to −48.69 ppm/℃ at S_T_ = 1000 °C, which agrees with the varying trend of apparent density. As we know, the τ*_f_* value is correlated with the phase composition, degree of densification and distortion of oxygen octahedra [36,37]. Thus, associating Figure 7d with Table 3, it is obvious that the increased τ*_f_* value is primarily determined by the enhancement in densification before S_T_ increases to 925 °C. At further increasing S_T_ with S_T_ > 925 °C, the low densification contributes to decreasing the τ*_f_* value. The τ*_f_* value exhibits an upward tendency owing to the decreased densification, which is due to the the abnormal grain growth caused by the higher sintering temperature.

## 4. Conclusions

In this work, the phase composition, microstructure, crystal structure and microwave dielectric properties of MCN ceramics were investigated as a function of the sintering temperature. MCN ceramics were confirmed to be multi-phase ceramics by XRD and Raman analysis. The SEM analysis showed that the dense microstructures appeared at 925 °C. As S_T_ increased from 875 °C to 925 °C, the grain growth was obvious, and the *Q* × *f* value, τ*_f_* value and ε_r_ increased from 23.230 GHz to 34.355 GHz, from −59.01 ppm/°C to −22.5 ppm/°C and form 16.12 to 17.15, respectively, and the dielectric losses and porosity declined form 4.740 × 10^−4^ to 3.436 × 10^−4^ and from 15.63% to 1.13%, respectively. However, when S_T_ was further increased with S_T_ > 925 °C, the *Q* × *f* value, τ*_f_* value and ε_r_ declined gradually, while the dielectric losses and porosity increased accordingly. Thus, the dielectric properties such as ε_r_, *Q* × *f* and τ*_f_* depended very strongly on the densification and microstructure of the samples, which was closely related to the sintering temperature. The sample prepared at the sintering temperature of 925 ℃ presented the largest values of *Q* × *f* value and apparent density, 34.355 GHz and 5.519 g/cm^3^, respectively. Correspondingly, the porosity decreased from 15.63% to 1.13% as the sintering temperature increased form 875 °C to 925 °C, and then increased with the further increased sintering temperature. The MCN ceramics prepared at the sintering temperature of 925 °C, and then annealed at 800 °C displayed dense microstructures, and possessed outstanding microwave dielectric properties of ε_r_ = 17.15, *Q* × *f* = 34.355 GHz, and τ*_f_* = −22.5 ppm/°C. These results imply that the MCN ceramics are suitable candidates for application in LTCC devices.

## Figures and Tables

**Table 1:** Crystal structure information of the MgNb_2_O_6_ (p1) and CuO (p2) after refinement.

S_T_ (°C)	Lattice Parameter									R_WP_	R_P_	χ^2^
	a_p1_ (Å)	b_p1_ (Å)	c_p1_ (Å)	V_p1_ (Å^3^)	W_p1_	a_p2_ (Å)	b_p2_ (Å)	c_p2_ (Å)	V_p2_ (Å^3^)			
875	14.1865	5.7032	5.0325	407.168	73.05%	4.6889	3.4198	5.1313	81.164	5.95%	4.57%	2.676
900	14.1827	5.7025	5.0306	406.855	77.43%	4.6889	3.4197	5.1291	81.110	5.95%	4.37%	3.678
925	14.1857	5.7024	5.0324	407.081	77.12%	4.6874	3.4171	5.1266	80.991	5.5%	3.94%	2.879
950	14.1750	5.6975	5.0289	406.146	76.58%	4.6880	3.4157	5.1253	80.941	5.37%	4.16%	2.679
975	14.1612	5.6911	5.0232	404.835	76.43%	4.6819	3.4068	5.1194	80.543	5.97%	4.51%	3.201
1000	14.1611	5.6914	5.0241	404.929	74.82%	4.6794	3.4087	5.1157	80.460	5.30%	4.11%	2.386

**Table 2:** The EDS results of MgCu_2_Nb_2_O_8_ ceramics corresponding to the abnormal grains.

Spot	Atom Fraction/(%)			
	Mg	Cu	Nb	O
A	11.81		22.38	65.81
B		52.12		47.88
C	11.93		22.66	65.41
D		50.87		49.13
E	12.06		23.71	64.23
F		51.59		48.41

**Table 3:** The apparent density, theory density, relative density and porosity of MgCu_2_Nb_2_O_8_ ceramics sintered at different temperatures.

S_T_ (°C)	ρ_apparent_ (g/cm^3^)	ρ_p1_ (g/cm^3^)	ρ_p2_ (g/cm^3^)	ρ_theory_ (g/cm^3^)	ρ_relative_ (%)	Porosity (%)
875	5.161	4.994	6.510	5.883	87.73	15.63
900	5.324	5.029	6.481	5.796	91.86	5.42
925	5.519	5.026	6.483	5.803	95.11	1.13
950	5.467	5.022	6.488	5.815	94.02	2.83
975	5.386	5.025	6.506	5.828	92.42	4.12
1000	5.192	5.021	6.517	5.865	88.53	8.67

## Data Availability

Not applicable.
